# Associations Between Neurological Diseases and Dioxin Exposure Originating from Agent Orange in Vietnam: A Narrative Review

**DOI:** 10.3390/toxics14050419

**Published:** 2026-05-11

**Authors:** Tai Pham-The, Hang Thi Nguyet Pham, William R. Folk, Nghi Ngoc Tran, Tuyet-Hanh Tran-Thi, Hisao Nishijo, Muneko Nishijo

**Affiliations:** 1 Biomedical and Pharmaceutical Research Centre, Vietnamese Military Medical University, Hanoi 12108, Vietnam; taithuy@kanazawa-med.ac.jp; 2 National Institute of Medicinal Materials, Hanoi 11018, Vietnam; nguyethangpt@nimm.org.vn; 3 School of Medicine, University of Missouri, Columbia, MO 65211, USA; folkw@missouri.edu; 4 Faculty of Clinical Medicine, Hanoi University of Public Health, Hanoi 11910, Vietnam; tnn@huph.edu.vn; 5 Department of Environmental Health, Hanoi University of Public Health, Hanoi 11910, Vietnam; tth2@huph.edu.vn; 6 Department of Sport and Health Sciences, Faculty of Human Sciences, University of East Asia, Shimonoseki 751-8503, Japan; 7 Department of Public Health, Kanazawa Medical University, Uchinada 920-0293, Japan

**Keywords:** Agent Orange, dioxin, neurological diseases, Vietnam

## Abstract

Now, half a century after the Vietnam War, Agent Orange exposure during the war is increasingly discussed as a risk factor for neurological diseases, particularly dementia and Alzheimer’s disease, among Korean and U.S. Vietnam War veterans. Emerging evidence supports associations between dioxin exposure originating from Agent Orange and alterations in brain morphology and function among Vietnamese residents, including children living in areas around former U.S. airbases exposed to 2,3,7,8-tetrachlorodibenzo-p-dioxin (TCDD) after the Vietnam War. This paper summarizes what is known about the effects of Agent Orange exposure in the context of neurological disorders, including Alzheimer’s disease, Parkinson’s disease, autism spectrum disorder, attention-deficit/hyperactivity disorder, and psychiatric disorders. Molecular biological studies have reported that TCDD may increase the risks of these neurological diseases by accelerating brain aging and inducing atypical neurodevelopment, partly mediated by the aryl hydrocarbon receptor. However, the effects of TCDD, which is a highly toxic contaminant of Agent Orange, as well as dioxin congeners other than TCDD, exhibit some inconsistencies. This review aims to provide new insights for mitigating the adverse neurological effects of dioxin exposure from Agent Orange, contributing to a healthier life for residents in Vietnam.

## 1. Introduction

**Figure 1:**
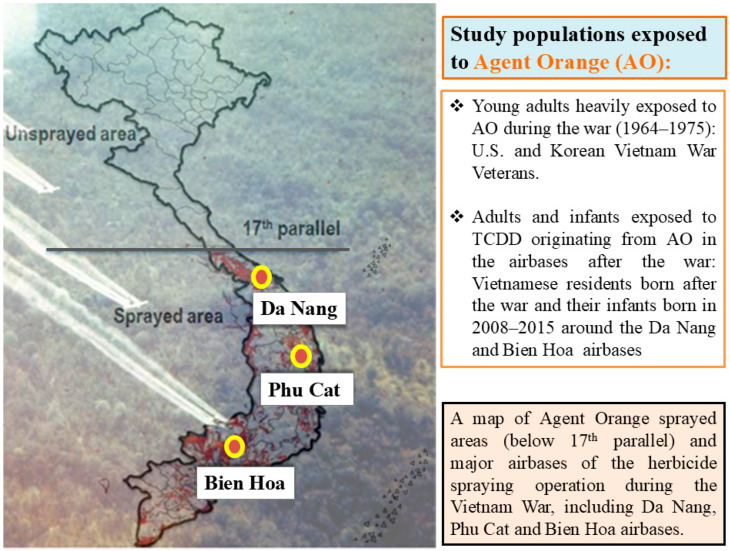
Major airbases that used Agent Orange spraying during the Vietnam War and study populations exposed to Agent Orange. TCDD, 2,3,7,8-tetrachlorodibenzo-p-dioxin.

In central and southern Vietnam, several military airbases, such as Da Nang, Phu Cat, and Bien Hoa, which the U.S. Army used for herbicide spraying campaigns during the Vietnam War were heavily contaminated with dioxins originating from dumped herbicides after the war, as well as spilled herbicides during mixing and loading works (Figure 1). These herbicides contained 2,4-dichlorophenoxyacetic acid (2,4-D) and 2,4,5-trichlorophenoxyacetic acid (2,4,5-T) with different mixing ratios. They were named Rainbow Agents because of the colorful bands painted on their containers. Agent Orange is the herbicide with the strongest impact on the environment and humans. It primarily contains 2,4-D and 2,4,5-T contaminated with 2,3,7,8-tetrachlorodibenzo-p-dioxin (TCDD) at a very high percentage, and it was used in large quantities during the Vietnam War for spraying [1].

During this war, approximately three million Americans served as military personnel in Vietnam (1964–1975) (Figure 1), 85% of whom were White males [2]. The Korean military also sent 320,000 military personnel to Vietnam, who served alongside the Americans from 1964 to 1973 [3] (Figure 1). Among them, approximately 1.5 million U.S. military personnel who served in South Vietnam from 1967 through 1969 were estimated to be the most heavily exposed to herbicides. In particular, the personnel who were assigned to aerial herbicide spraying in Operation Ranch Hand and Army Chemical Corps-trained soldiers who sprayed herbicides on the ground were believed to be at the highest risk of Agent Orange exposure. However, many more military personnel engaged in small-scale herbicide spraying [2], making it difficult to estimate the population exposed to Agent Orange and therefore at consequent health risk.

To review the scientific evidence on the possible health effects of Agent Orange exposure, the U.S. government called the National Academy of Sciences in 1991, and the first review book was published to report possible diseases associated with Agent Orange exposure in 1994 [4]. In the 1996 report [5], the committee updated their evidence and reported five diseases with sufficient evidence showing an association with Agent Orange and/or TCDD, including soft-tissue sarcoma, non-Hodgkin’s lymphoma, Hodgkin’s disease, chloracne, and porphyria cutanea tarda. However, the committee concluded that there was limited suggestive evidence of an association between exposure to the herbicides used in Vietnam and spina bifida in offspring [5], although neural tube defects causing spina bifida or anencephaly were increased among the offspring of Operation Ranch Hand veterans [6]. Moreover, a central nervous system anomaly was more frequently observed among the children of Vietnam veterans than among the children of non-Vietnam veterans [7].

Regarding neurological diseases in adults, the committee indicated that brain diseases with motor/coordination dysfunction, such as Parkinson’s disease (PD), should be investigated after a long follow-up, although they found no definitive studies to show associations of such diseases with Agent Orange exposure. However, associations of agricultural chemicals, such as herbicides and pesticides, with PD have been suggested [8]. The increased PD prevalence, especially young-onset PD, has been investigated among U.S. Vietnam War veterans [5]. Moreover, one study evaluating acute and subacute (transient) peripheral neuropathy demonstrated limited suggestive evidence of an association with Agent Orange exposure, primarily because of symptom self-reporting, although the study was a large-scale case–control study [9].

More than 30 years after the Vietnam War ended, Yi et al. (2014) [10] suggested increased morbidity of various diseases and increasing systemic atrophy affecting the nervous system, including spinal muscular atrophy, Alzheimer’s disease (AD), and peripheral polyneuropathies in Korean veterans exposed to Agent Orange. They also reported a higher prevalence of psychotic diseases in the high-exposure group of the self-report study [3]. Increased plasma amyloid-beta (Aβ) oligomer levels indicating AD [11] and brain atrophy progression were examined in magnetic resonance imaging (MRI) studies among Korean Vietnam War veterans exposed to Agent Orange [12]. Among U.S. Vietnam War veterans, an increased prevalence of dementia has been reported [13], and Agent Orange exposure is increasingly discussed as a risk factor for neurological diseases, particularly brain atrophy and AD.

Several former U.S. military airbases, located in central and southern Vietnam, remained contaminated with Agent Orange and other herbicides used during the Vietnam War, even 40 years after the war had ended (Figure 1). The levels of TCDD, which is a specific dioxin congener to Agent Orange, in the breast milk of mothers residing near Da Nang and Bien Hoa airbases were found to be 3–4 times higher than those in unsprayed areas [14], suggesting increased health risks among the infants and children around these airbases. We followed up with these birth cohorts and found significant adverse effects of TCDD on their neurodevelopment from 4 or 6 months to 9 years of age. In particular, at 3 years of age, increased traits of autism indicated by specific symptoms and gaze behavior were found in children and were associated with higher perinatal TCDD exposure [15]. At 8 years of age, increased attention-deficit hyperactivity disorder (ADHD) behaviors in girls [16] and learning difficulty (LD), especially for reading, in boys were observed among children exposed to higher levels of TCDD [17]. These findings indicate that TCDD exposure originating from Agent Orange may influence brain development during the perinatal period, resulting in neurodevelopmental deficits, such as autism spectrum disorder (ASD) and ADHD. The effects of TCDD on infant neurodevelopment are also supported by study results, among Vietnamese men, of fathers from one of our birth cohorts in Bien Hoa, showing alterations in brain morphology and function associated with estimated Agent Orange exposure during the perinatal period [18,19,20].

In this review, we summarize the key findings on the effects of Agent Orange in the context of neurological disorders, including AD, PD, psychiatric disorders, and neurodevelopmental disorders, such as ASD and ADHD.

## 2. Materials and Methods

This narrative review focused on the epidemiological studies of neurological diseases and psychiatric symptoms resulting from the neurotoxic effects of Agent Orange exposure during the Vietnam War or TCDD exposure originating from Agent Orange at former U.S. airbases in Vietnam after the war. The methodology involved a comprehensive literature search of original research, substantial reviews, or government reports published in English from 1988 to 2025.

To identify the relevant literature, we searched electronic databases, such as PubMed, ScienceDirect, and Google Scholar, using combinations of pertinent key terms, including (a) Parkinson’s disease; dementia and/or Alzheimer’s Disease; neuropathy; psychological diseases and/or symptoms; neurodevelopmental disorders, including ASD and/or ADHD; (b) dioxins, 2,3,7,8-tetrachlorodibenzo-p-dioxin (TCDD), and Agent Orange; (c) Vietnam; Vietnam War.

After screening titles and abstracts for relevance, and the full texts of eligible studies, only epidemiological studies with reliable diagnostic criteria of targeted neurological diseases or symptoms specific to the diseases, were reviewed. Studies were excluded if they showed no clear exposure assessment method to Agent Orange or dioxins. A narrative overview of the selected high-quality evidence, indicated by increases in incidence or prevalence of the neurological diseases in adults, increased standardized scores of rating scale for the neurodevelopmental disorders, and increased biomarkers specific to the diseases (e.g., Aβ for Alzheimer’s Disease), were provided along with future directions for the early detection of key neurological diseases in the population at high risk of Agent Orange exposure in this review.

## 3. Results

Nearly 10 years after the Vietnam War ended, several large-scale studies investigated veterans’ health and reported neurological effects caused by Agent Orange exposure, such as a higher prevalence of neuropathy or increased psychotic symptoms, such as depression and anxiety, among Korean [3,21] and U.S. Vietnam War veterans [22,23,24]. After longer follow-up, increased prevalence of neurodegenerative diseases, such as AD, was reported [12,13], suggesting poor neurological outcomes more than 30 years after cessation of Agent Orange exposure. In the Vietnamese population, birth cohort studies evaluating the effects of TCDD on neurodevelopment in children have been conducted [15,16,17,25,26,27,28,29,30,31,32,33]. Moreover, studies on their fathers have been performed to investigate the associations between brain regional volume and function and perinatal Agent Orange exposure of fathers suggesting ASD in adults, as well as associations with adulthood dioxin exposure indicated by blood levels [18,19,20].

The key studies identifying associations between neurological diseases or symptoms and Agent Orange exposure during adulthood or the perinatal period are listed in Table 1 and Table 2, respectively, and a narrative overview of evidence for each neurological disease was provided according to different exposure duration, adulthood exposure, or perinatal exposure.

### 3.1. PD

When the possible diseases associated with Agent Orange exposure were reviewed by the National Academy of Sciences of the U.S., the potential effects of Agent Orange on the brain and neurological systems were suggested by Semchuk et al. (1993) [34]. The occupational use of herbicides was one of the strongest predictors of neurodegenerative diseases, such as PD. Butterfield et al. (1993) [35] also indicated occupational and environmental factors associated with PD, particularly early-onset PD.

**Table 1:** Key studies reporting suspected associations of neurological diseases with adulthood Agent Orange exposure.

Neurological Diseases	Targeted Population	Indicators	References
PD	U.S. veterans	Reviewing previous studies	Semchuk et al. (1993) [34]
	Korean veterans	Brain imaging using dopamine transporter	Yang et al. (2016) [36]
	Korean veterans	PD incidence	Song et al. (2023) [37]
Dementia and AD	Korean veterans	Progression of brain atrophy using MRIs	Lee et al. (2022) [12]
	Korean veterans	Increased adjusted odds ratios for AD	Yi et al. (2014) [10]
	Korean veterans	Increased Aβ oligomer levels in plasma	Yang et al. (2018) [11]
	U.S. veterans	Increased prevalence of dementia	Martinez et al. (2021) [13]
Neuropathy	U.S. veterans	Increased prevalence of peripheral neuropathy	Yi et al. (2013) [3]; (2014) [10]
Psychiatric diseases and symptoms	U.S. veterans	Higher prevalence of depression, substance use disorder, and post-traumatic stress disorder	Martinez et al. (2021) [13]
	Korean veterans	Higher risk ratio of psychotic diseases	Yi et al. (2013) [3]
Altered brain regional volume	Vietnamese residents	Decreased some brain regional volume associated with increased blood dioxin levels	Vu et al. (2021) [18]; Nguyen et al. (2025) [20]

PD, Parkinson’s disease; AD, Alzheimer’s Disease.

Yang et al. (2016) [36] examined the clinical PD symptoms such as resting tremor and rigidity and dopamine transporter (DAT) positron emission tomography (PET) findings between Korean patients exposed and not exposed to Agent Orange (N = 143, and N = 500, respectively), and reported that PD patients with Agent Orange showed significantly higher asymmetry of motor symptoms and lower DAT uptake in all areas of the basal ganglia in the midbrain, particularly in the putamen (*p* = 0.000), compared with patients without exposure. These results suggest clinical characteristics of PD in the patients exposed to Agent Orange, although authors analyzed the data without adjusting confounding factors.

A retrospective cohort study was conducted by Song et al. (2023) [37] among Korean Vietnam War veterans (N = 37,246; 100% male; age, 65.6 ± 8.1 years) using data from medical records obtained from the Veterans Health Service Medical Center over a 12-year period (2009–2020). They showed a 1.31 times higher PD risk among veterans with Agent Orange exposure alone, and a 1.68 times higher adjusted odds ratio (OR) among veterans with Agent Orange exposure (OR [95% CI], 1.68 [1.36, 2.08]) and drug use associated with drug-induced Parkinsonism (DIP).

### 3.2. Dementia and AD

#### 3.2.1. Epidemiological Studies of Korean Vietnam War Veterans

In Korea, a prospective cohort study [12] and a retrospective cohort study [10] of Vietnam War veterans suggested that Agent Orange exposure may promote brain atrophy, which may increase dementia risk. Lee et al. [12] investigated the effects of dioxins on brain atrophy progression among 241 pairs of Korean veterans exposed to Agent Orange (mean age, 65.5 years) and non-exposed controls (mean age, 65.7 years), matched with age at initial examination and interval between the two MRI examinations. The subjects in both the exposed and control groups underwent brain T1-weighted MRI twice, with a mean interval of around 1180 days. Voxel-based morphometry analysis of the MRI scans indicated significantly greater progression of brain atrophy in the bilateral frontal and temporal lobes in the exposed group than in the control group. In particular, brain atrophy was evident in the ventrolateral prefrontal area of the frontal lobe and whole areas of the temporal lobe in the exposed group (threshold-free cluster enhancement-corrected *p* < 0.05).

Yi et al. [10] investigated the prevalence of diseases, including those of the nervous system, associated with Agent Orange exposure between 1961 and 1971 among 111,726 Korean Vietnam War veterans over a 5-year observation period starting in 2000. Exposure to Agent Orange was evaluated using a geographic information system-grounded model. The results indicated increased adjusted ORs for various neurological diseases, including AD (OR [95% CI], 1.64 [1.12, 2.41]), spinal muscular atrophy, and peripheral polyneuropathies in the high Agent Orange exposure group compared with the low-exposure group.

Consistently, in Korean Vietnam War veterans aged 50–90 years in 2015–2016, plasma levels of Aβ oligomers, which are toxic and known to induce neuronal and synaptic damage in the brain leading to AD [38,39], were higher in patients with AD with Agent Orange exposure (N = 48) than in those with AD without exposure (N = 66) (*p* < 0.0003), and in controls without AD symptoms (N = 60) who belonged to same categories of age and education (*p* < 0.0001) [11], although statistical tests were performed without adjusting confounding factors. These findings suggest that Agent Orange exposure may affect the pathology of AD by increasing Aβ oligomers in the brain, which is reflected in increased plasma Aβ oligomer levels.

**Table 2:** Key studies reporting suspected associations of neurodevelopmental disorders with perinatal Agent Orange exposure.

Suspected Diseases	Targeted Population	Indicators	References
Neurodevelopmental deficits	DaNang birth cohort in Vietnam	Lower Bayley III scores at 4 months and 3 years of age	Tai et al. (2013) [25]; Nishijo et al. (2014) [15]
ASD	DaNang birth cohort in Vietnam	Higher scores of Autism Spectrum Rating Scale (ASRS)	Nishijo et al. (2014) [15]
Neurodevelopmental deficits	DaNang birth cohort in Vietnam	Lower marginal Bayley III scores through the first 3 years of life	Tai et al. (2016) [26]
Neurodevelopmental deficits; poor cognitive ability and coordination motor skills	DaNang birth cohort in Vietnam	Lower non-verbal index (NVI) of KABC-II and MABC-2 scores at 5 years of age	Tran et al. (2016) [27]
ADHD	DaNang birth cohort in Vietnam	Higher ADHD rating scale at 8 years of age	Pham-The et al. (2022) [16]
LD	DaNang birth cohort in Vietnam	Higher score of difficulty in learning related to language	Pham The et al. (2020) [17]
ASD	DaNang birth cohort in Vietnam	Reduced EEG power of theta rhythms during hand movements (Mirror neuron activity)	Vu et al. (2021) [28]
High risk of ASD	Bien Hoa birth cohort in Vietnam	Lower expressive language at 2 years of age	Pham et al. (2019) [29]
ASD	Bien Hoa birth cohort in Vietnam	Atypical gaze behaviors; lower fixation density on faces	Pham et al. (2022) [30]
High risk of ASD	Bien Hoa birth cohort in Vietnam	Altered relative EEG power in the sleep stage of newborns leading to poor language development	Nghiem et al. (2019) [31]
ASD	Bien Hoa birth cohort in Vietnam	Atypical gaze behavior and higher ASRS scores	Thao et al. (2023) [32]
ASD suspected in adults	Fathers from Bien Hoa birth cohort	Increased brain regional volume and social anxiety in men exposed to Agent Orange during their perinatal period	Vu et al. (2021) [18]; Vu et al. (2023) [19]; Nguyen et al. (2025) [20]
ASD	Children of immigrant mothers born in Vietnam from birth cohort in Finland	Increased risk of childhood autism diagnosis	Lehti et al. (2013) [40]

ASD, autism spectrum disorder; ADHD, attention-deficit hyperactivity disorder; Bayley III, Bayley Scales of Infant and Toddler Development, third edition; KABC-II, Kaufman Assessment Battery for Children, second edition; LD, learning difficulty; MABC-2, Movement Assessment Battery for Children, second edition.

#### 3.2.2. A Retrospective Cohort Study of U.S. Vietnam War Veterans

Martinez et al. [13] analyzed the adjusted risk ratios of Agent Orange exposure during the Vietnam War for dementia diagnosis by comparing the prevalence of dementia between U.S. veterans with and without exposure to Agent Orange. Among more than 300,000 veterans who engaged in military service in Vietnam (1964–1975), the authors sampled targeted subjects from the Veterans Health Administration (VHA)’s records of veterans who received VHA care between 2001 and 2015, and detected those diagnosed with dementia. The participants were mostly male (98%) and had a mean age of 62 years. After adjusting for demographic variables and comorbidities, the risk ratio for dementia among U.S. veterans with Agent Orange exposure was around twice that of veterans without Agent Orange exposure (OR [95% CI], 1.68 [1.59, 1.77]). Furthermore, the mean age ± standard deviation (SD) at dementia diagnosis was 1.25 years younger among veterans with Agent Orange exposure than among those without exposure (67.5 ± 7.0 vs. 68.8 ± 8.0 years). These findings indicate that Agent Orange exposure during the Vietnam War significantly increased the prevalence and earlier-onset of dementia, including AD among U.S. veterans, as well as Korean veterans.

### 3.3. Other Neurological Diseases and Psychiatric Symptoms

#### 3.3.1. Neuropathy

Yi et al. (2013) [3] investigated the associations between the prevalence of neuromuscular diseases and levels of Agent Orange exposure among Korean Vietnam War veterans, reporting a significantly increased OR (OR [95% CI], 1.07 [1.02, 1.12]) for peripheral neuropathy in the high-exposure group compared with the low-exposure group. They also investigated the prevalence of peripheral polyneuropathies using Korea National Health Insurance’s claims data, again reporting a significantly increased OR (OR [95% CI], 1.09 [1.04, 1.13]) [10].

De la Monte and Goel (2022) [2] reviewed these Korean studies to investigate the prevalence of peripheral neuropathy among veterans exposed to Agent Orange. Highly exposed veterans showed a significantly higher prevalence of symmetric peripheral neuropathy than controls or veterans exposed to lower levels. The rate of diabetes among exposed veterans was high; 87.5% of veterans who were highly exposed to Agent Orange and diagnosed with peripheral neuropathy had diabetes mellitus. These findings suggest that Agent Orange exposure at high levels may cause both peripheral neuropathy and diabetes mellitus, which may interactively aggravate neuropathy.

#### 3.3.2. Psychiatric Symptoms

Martinez et al. (2021) [13] compared the prevalence of comorbid mental health conditions between U.S. Vietnam War veterans (N = 38,121) who were heavily exposed to Agent Orange and matched controls (N = 23,472). They observed a significantly higher prevalence of depression (13.7% vs. 8.4%), substance use disorder (7.6% vs. 5.3%), and post-traumatic stress disorder (10.0% vs. 3.2%) among the group exposed to Agent Orange. A higher prevalence of psychiatric symptoms, such as depression and anxiety symptoms, was also reported by Stellman et al. (1988) [23] and Michalek et al. (2003) [24], among Vietnam War veterans in the early period after the end of the war. Particularly, Operation Ranch Hand veterans with higher dioxin levels (N = 1109) showed difficulties with anxiety, somatization, depression, and a denial of psychological factors, compared with air force veterans not involved in herbicide spraying (N = 1493) [24]. In addition, significant but only slightly increased adjusted OR of psychotic diseases was observed (OR [95% CI], 1.07 [1.00, 1.14]) among Korean veterans in the group with high Agent Orange exposure, than in the group with low exposure, although exposure was based on self-reporting [3].

#### 3.3.3. Altered Brain Regional Volumes Analyzed by Brain MRIs

Vu et al. (2021) [18] studied 32 Vietnamese men who were fathers of infants from a birth cohort residing nearby the Bien Hoa airbase, recruited in 2015 (Bien Hoa birth cohort 2015) and reported neurodevelopmental deficits associated with increased perinatal TCDD exposure originating from Agent Orange contamination at the Bien Hoa airbase. Blood or serum dioxin levels sampled during the study were used as a marker of exposure in adulthood. All subjects underwent brain MRIs to analyze regional brain cortical gray matter volumes using voxel-based morphometry. Blood TCDD levels were inversely correlated with brain regional volume in the medial temporal pole and fusiform gyrus (FDR-corrected at *p* < 0.05), while blood levels of toxic equivalency (TEQ)-polychlorinated dibenzodioxins (PCDDs) were inversely correlated with volume in the medial temporal pole (FDR-corrected at *p* < 0.05) [18]. In addition, significant inverse associations between biological toxic equivalency (BEQ) in the sera and brain volumes in several right temporal gyri in men (exposed and unexposed) were reported (standardized β = −0.698, *p* < 0.05) [20], suggesting that dioxin exposure after brain development (during late childhood and adulthood) may decrease cortical gray matter volumes in gyri that contribute to cognitive ability, possibly leading to cognitive dysfunction and dementia in old age.

### 3.4. Neurodevelopmental Disorders: ASD, ADHD, and LD

#### 3.4.1. Neurodevelopmental Disorders in Children

##### Danang Birth Cohort Studies in Vietnam

A series of follow-up studies involving infants and children from the Da Nang birth cohort identified adverse effects of perinatal dioxin exposure indicated by concentrations of PCDD/Fs congeners, including TCDD and TEQ-PCDD/Fs, in their maternal breast milk on neurodevelopment using the Bayley Scales of Infant and Toddler Development, third edition (Bayley III) at 4 months to 3 years of age [15,25,26]. In all 4-month-old infants, significantly increased cognitive and fine motor scores (effect size [ES] = 0.44, *p* = 0.009, and ES = 0.46, *p* = 0.030, respectively) were found in the high TEQ-PCDD/Fs exposure group (≥17.6 pg-TEQ/g lipid in maternal breast milk) compared with the low-exposure group (<7.4 pg-TEQ/g lipid) [24]. In only boys, expressive language score was also significantly lower (ES = 0.20, *p* < 0.05) in the high TEQ-PCDD/Fs group than that in low-exposure group [25].

Increased autistic traits indicated by higher Autism Spectrum Rating Scale (ASRS) scores were associated with higher perinatal TCDD exposure (≥3.5 pg/g lipid) in 3-year-old children, particularly boys (Diagnostic and Statistical Manual of Mental Disorders, Fourth Edition, Text Revision [DSM-IV-TR] score: 61.5 vs. 56.1, ES = 0.11, *p* = 0.002) [15]. However, no significant relationship between autistic traits and TEQ values of polychlorinated dibenzodioxins and polychlorinated dibenzofurans (TEQ-PCDD/Fs) was found among these children, although boys with high levels of TEQ-PCDD/Fs showed poor language and motor development through the first 3 years of life [26]. These findings suggest that dioxin exposure may influence the neurodevelopment of children, but TCDD may specifically influence development of the social brain and increase autistic traits among exposed children.

At 5 years of age, cognitive ability was assessed using the Kaufman Assessment Battery for Children, second edition (KABC-II), and coordination motor ability was examined using the Movement Assessment Battery for Children, second edition (MABC-2). Boys showed lower non-verbal index (NVI) scores (ES = 0.083, *p* = 0.010) and pattern reasoning subscale scores (ES = 0.052, *p* = 0.041) in the KABC-II, in the higher TCDD group (≥2.5 pg/g lipid), whereas MABC-2 scores were significantly lower in the high TEQ-PCDD/Fs (≥17.6 pg TEQ/g lipid) group (ES = 0.107, *p* = 0.018) [27]. These results suggest that dioxin influences neuronal functions only in boys, and TCDD exposure was associated with lower cognitive ability which can be often found in children with ASD, whereas TEQ-PCDD/Fs exposure was associated with motor function at 5 years of age, as well as at 3 years of age.

Another study showed that at 8 years of age, significantly increased ADHD behaviors, particularly hyperactivity/impulsivity, were observed only among girls with perinatal exposure to high levels of TCDD compared with girls exposed to low levels (adjusted mean score [95% CI], 56.3 [41.7, 71.0] vs. 39.1 [23.8, 45.4]) [16]. At the same age, boys with high TCDD exposure showed LD for language with higher Colorado LD Questionnaire reading scores (ES = 0.08, *p* = 0.01), another neurodevelopmental disorder demonstrating particular difficulty in learning related to language [17], although girls did not show increased LD scores associated with perinatal dioxin exposure. These findings suggest that perinatal TCDD exposure impacts social–emotional cognitive function, leading to sex-specific neurodevelopmental disorders, including LD in boys and ADHD in girls.

In addition, at 9 years of age, we investigated the effects of perinatal dioxin exposure on the activity of the mirror neuron system in the brain, indicated by reduced electroencephalography (EEG) power of theta rhythms during hand movements. We reported that decreased theta EEG power was significantly lower (ES = 0.165, *p* = 0.001) among girls with high TCDD exposure (≥3.0 pg/g lipid) and among boys (ES = 0.087, *p* = 0.020) with high TEQ-PCDD/Fs (≥17.6 pg TEQ/g lipid) [28]. These results suggest that the mirror neuron system in the brain might be impaired in children exposed to high levels of dioxins during the perinatal period, as well as in children with ASD, although impairment was associated with TEQ-PCDD/Fs.

##### Bien Hoa Birth Cohort Studies in Vietnam

In the area around Bien Hoa airbase, we recruited 210 mother–newborn pairs living in 10 communes close to the Bien Hoa airbase in 2012 (Bien Hoa birth cohort 2012) and who reported higher mean concentrations of TCDD in breast milk (2.6 pg/g lipid for primiparous mothers and 2.2 pg/g lipid for multiparous mothers) compared with samples of unexposed mothers in Hanoi (a control birth cohort) and mothers in Da Nang birth cohort. In 2015, we additionally collected 78 mother–child pairs as the second birth cohort in the Bien Hoa region (Bien Hoa cohort 2015). We combined the Bien Hoa 2012, Bien Hoa 2015, and Hanoi cohorts, and reported significantly decreased expressive language scores at 2 years of age among boys with high TCDD exposure (≥5.5 pg/g lipid) compared with boys with low exposure (<1.8 pg/g lipid) (adjusted mean score [95% CI], 6.5 [4.8, 8.1] in the high TCDD group vs. 8.8 in the low TCDD group) [29]. At 3 years of age, girls from the Bien Hoa 2012 cohort showed atypical gaze behaviors, such as lower fixation density on faces (adjusted mean score [95% CI], 25.5 [12.7, 38.3] vs. 43.4 [35.8, 50.9], *p* = 0.025), which were inversely associated with perinatal TCDD exposure and inversely correlated with the scores of the social communication scale, one of the ASRS subscales (standardized β value = −0.290, *p* = 0.038) [30]. These poor gaze behaviors suggest that children from the Bien Hoa cohort, as well as children from the Da Nang cohort, may have social cognitive deficits similar to ASD associated with perinatal TCDD exposure.

On the second day after birth, during sleep time, we recorded neonatal EEG among newborn infants recruited to the Bien Hoa cohort in 2015, showing that TCDD exposure altered relative EEG power in the active sleep stage, associated with an increase in Bayley III language scores at 2 years of age [31]. We also reported decreased relative EEG power in the quiet sleep stage associated with increasing TCDD exposure, which was associated with poor gaze behavior at 2 years of age, as shown by a shorter fixation duration on the face of a child talking in videos. Gaze behavior was also investigated among 2-year-old children from Bien Hoa cohort 2015 during viewing dynamic social stimuli, and reduced face fixation duration was found among boys in the higher TCDD exposure group compared with the lower exposure group (*p* < 0.047). When they reached 3 years of age, higher ASRS scores indicating increased autistic traits were found in the higher perinatal TCDD exposure group (mean [standard error] DSM-IV-TR score: 61.2 [1.7] vs. 55.5 [0.9], *p* = 0.006) for both sexes [32]. These findings suggest that prenatal TCDD exposure affects neuronal activity and may lead to poor communication ability in childhood, which is often found in children with ASD.

At 5 years of age, KABC-II and MABC-2 scores were compared between an exposed group of 185 children from the Bien Hoa 2012 cohort and an unexposed control group of 104 children from the Ha Dong cohort in Hanoi City, and significant differences were observed [33]. Among boys, significantly lower test scores on the triangle subscale (10.7 vs. 12.4, *p* = 0.005) of KABC-II and the balance and total movement scales (7.4 vs. 10.3, *p* < 0.001) of MABC-2 were associated with increasing TCDD exposure, while lower test scores on the hand movement subscale (9.6 vs. 11.3, *p* = 0.003) of KABC-II and the balance subscale (9.1 vs. 10.7, *p* = 0.050) of MABC-2 were associated with TEQ-PCDD/Fs among girls. These results suggest that perinatal dioxin exposure appears to have a long-term impact on neurodevelopment in children under 6 years old, of both sexes, in Bien Hoa, which is the area most contaminated by dioxins from Agent Orange in Vietnam [41,42], whereas neurodevelopmental impact was observed mostly in boys in Da Nang, contaminated with other kinds of herbicides as well as Agent Orange.

##### Birth Cohort Study for Immigrants from Vietnam in Other Countries

In Finland, Lehti et al. (2013) [40] conducted a nested case–control study based on a national birth cohort to clarify if immigrant mothers born outside Finland may have an increased risk of having a child with ASD. The results showed an increased risk of childhood autism among families where the mother was born in Vietnam and exposed to Agent Orange, suggesting increased risk of ASD among their offspring infants, which was consistently reported in the birth cohort studies in Vietnam [15,30,32].

Taken together, the results of the epidemiological studies in the areas around former U.S. airbases in Da Nang and Bien Hoa, Vietnam, illustrate the neurodevelopmental impact of perinatal dioxin exposure from Agent Orange and the presence of dioxins in breast milk, increasing neurodevelopmental disorders, such as ASD, ADHD, and LD, among children.

#### 3.4.2. Neurodevelopmental Disorders in Adults

In the neurological studies on fathers from the Bien Hoa birth cohort [18,19,20], the effects of perinatal dioxin exposure estimated by their mothers’ residency in a dioxin-contaminated area on brain regional volume and function was investigated, as well as effects of dioxin exposure during adulthood, indicated by blood dioxin levels described in Section 3.3.3. of the present review. Fathers with perinatal dioxin exposure (N = 12) showed significantly larger global brain gray matter volume compared with fathers without exposure after adjusting confounding factors (adjusted mean score [95% CI], 646.5 [624.6, 668.4] vs. 609.8 [590.5, 629.2], *p* < 0.05) [17] and matched unexposed controls (N = 10, *p* < 0.05) [19]. Particularly in the temporal lobe, brain regional volume was increased in fathers with perinatal exposure after adjusting confounding factors and increased brain volumes in the superior temporal gyrus (*p* < 0.05) and temporal pole (*p* < 0.01), which was contributed to enlargement of the temporal lobe associated with perinatal exposure (*p* < 0.05) [19]. The volume of the left inferior frontal gyrus pars orbitalis was significantly lower in men with perinatal dioxin exposure (FDR-corrected at *p* < 0.05) [18]. Social–emotional subscale scores on the social anxiety scale in men with perinatal dioxin exposure were significantly higher than those of men without perinatal exposure (*p* < 0.05) or the unexposed controls (*p* < 0.05) [19,20] after adjusting relevant factors including education and family income, indicating dioxin effects on social–emotional behavior, as well as brain morphology. These results suggest that dioxin exposure may increase the global and regional volumes of the temporal lobe, particularly the volumes of the regions which contribute to social and behavioral deficits in individuals with autism. Increased autistic traits (ASD symptoms) associated with perinatal exposure in birth cohort studies were also indicated in adults exposed to Agent Orange during fetal and infantile period.

## 4. Discussion

### 4.1. PD and Dementia Including AD

In a 12-year follow-up from 2009 to 2020, Korean Vietnam War veterans [37] showed a higher incidence of PD, particularly in veterans who used DIP-risk drugs, suggesting that the significant association of Agent Orange with PD might be visible only in studies with carefully adjusted co-factors, such as the DIP-risk drug. However, an increased risk of PD associated with Agent Orange exposure has not been reported among U.S. Vietnam War veterans, suggesting that Agent Orange exposure may not trigger PD on its own, but enhance the effect to induce PD of other factors such as DIP-risk drug.

In a large-scale incidence study in U.S.A. with a 14-year follow-up of Vietnam War veterans [13], significantly increased prevalence risk for dementia, including AD, was found among U.S. veterans with Agent Orange exposure. Also, among Korean Vietnam War veterans, Lee et al. (2024) [43] reported significantly increased risks of all types of dementia, including AD, vascular dementia, and unspecified dementia. In the clinical studies for Korean veterans, brain atrophy progression, particularly in the bilateral frontal and temporal lobes [12], and increased plasma Aβ oligomer levels were also reported [11], suggesting that Agent Orange exposure may increase the risk of dementia, including AD.

Despite these findings, the studies to investigate associations between serum dioxin levels and neurophysiological function indicating dose–effect relationships are limited. Barrett et al. (2001) [44] found significant associations between exposure categories based on serum total dioxin concentrations and cognitive disturbances among Operation Ranch Hand veterans approximately 20 years after the Vietnam War. Pelclova et al. (2018) [45] reported that current serum TCDD levels, examined long after retirement, were not associated with neuropsychological disturbances detected by single-photon emission computed tomography among herbicide production workers in Czechoslovakia, suggesting that their brain damage and neurophysiological deficits may have been induced by initial TCDD exposure and persisted long after the exposure.

Dioxins included in Agent Orange and other herbicides are the most powerful ligand of the aryl hydrocarbon receptor, which is believed to mediate the effects of dioxins on human health. Choudhary et al. (2020) [46] reviewed the role of the aryl hydrocarbon receptor in PD and AD as a mediator of the expression of several genes encoding the protein components of the ubiquitin proteasome pathway, specifically, Ubch7, an E2 ubiquitin enzyme partner of parkin [47,48]. Parkin is an E3 ligase catalyzing proteins for ubiquitination, and it plays an important role in the occurrence of PD [49,50]. In patients with AD, increased aryl hydrocarbon receptor expression in astrocytes compared with expression in healthy aged adults was observed at any age [51], suggesting that the aryl hydrocarbon receptor is implicated in the aging process of the brain and may be involved in the development of AD through its effects on astrocytes. Therefore, dioxin congeners contaminated in Agent Orange may increase aryl hydrocarbon receptor expression in the neurons or astrocytes and are partly involved in inducing the development of PD or AD.

Aryl hydrocarbon receptor signaling pathways also lead to pathological processes, including cellular oxidative stress resulting from the generation of reactive oxygen species; inflammation; the production of neurotoxic molecules, such as N-methyl-D-aspartate; and epigenetic alterations [51,52,53,54,55,56]. Interestingly, it has been reported that Aβ activates the aryl hydrocarbon receptor, which leads to the enhancement of tau phosphorylation and neuronal damage through downregulation of the Wnt/β-catenin signaling pathway [57], while persistent environmental chemical exposure may increase Aβ levels in the brain [58]. These findings suggest that herbicide exposure during the Vietnam War may promote Aβ-associated brain aging, leading to dementia, and particularly AD.

Moreover, recently, mitochondrial dysfunction is suggested as one of the most significant factors associated with AD caused by neuroinflammation and oxidative stress [59], which can be induced by exposure to various organic environmental chemicals including TCDD and disturb mitochondrial morphology, and the functions of neuronal cells [60], other than the aryl hydrocarbon receptor signaling pathway, need to be investigated in the near future.

### 4.2. Neuropathy and Psychiatric Symptoms

Depression, post-traumatic stress disorder, and anxiety are the most commonly reported psychiatric symptoms in studies evaluating Vietnam War veterans [22,23,24], suggesting poor mental health outcomes associated with Agent Orange exposure. However, as excessive mental strain from military service during the war is a powerful cause of psychiatric symptoms, the associations between Agent Orange exposure and psychiatric symptoms cannot be easily concluded. Also, in these studies, information was collected only from self-reporting questionnaires for psychiatric symptoms, and no objective index of symptoms was used.

In a German occupational cohort exposed to polychlorinated biphenyls (PCB), urinary homo-vanillic acid, which is a urinary dopamine metabolite indicating altered dopamine levels in the brain, decreased with an increase in total PCB longitudinally, and it was also associated with increased depressive symptoms 1 year later [61]. In an animal experimental study, female mice with a higher prevalence of depression compared with males were orally exposed to TCDD at a relatively lower dose and showed depression-like behavior, but no clear weight loss [62]. These findings suggest that dioxin exposure may worsen depressive symptoms but not be a primary cause of major depressive disorder.

In the general population, a large-scale Swedish cohort study reported that higher levels of leukocytes, haptoglobin, and C-reactive protein, and a lower level of immunoglobulin G, increased the risk of psychiatric disorders, such as anxiety and depression [63]. In an Iranian population study, increased leukocyte count and higher red cell distribution width were associated with more severe depression and anxiety symptoms in men and both sexes, respectively [64]. These studies suggest that dopamine and inflammatory biomarkers may provide useful information about the presence of psychiatric diseases. In the future, measurement of these biomarkers along with questionnaire surveys is recommended for evaluating depression and anxiety symptoms in the population exposed to Agent Orange.

### 4.3. Neurodevelopmental Disorders: ASD, ADHD, and LD

Several birth cohort studies in the areas around the contaminated Da Nang and Bien Hoa airbases in Vietnam have suggested effects of perinatal TCDD exposure originating from Agent Orange on the developing brain, suggesting an increased prevalence of childhood neurodevelopmental disorders, particularly, ADHD in girls, LD in boys, and ASD in both sexes [16]. In a nested case–control study based on a national birth cohort in Finland, Lehti et al. (2013) [40] also reported an increased risk of childhood autism among families in which the mother was born in Vietnam, suspecting that perinatal dioxin exposure may increase the risk of ASD among the offspring of Vietnamese mothers.

Moreover, fathers from the Bien Hoa cohort with perinatal Agent Orange exposure showed enlargement of the brain volume and social–emotional deficit [18,19], which are the most typical symptoms of ASD, suggesting increased risk for adulthood ASD in middle-aged offspring of mothers who had lived in the heavily Agent Orange-sprayed areas in Vietnam during pregnancy and after giving birth.

In a murine study, excessive aryl hydrocarbon receptor activation by neuronal transfection of constitutively active aryl hydrocarbon receptor vector plasmids via in utero electroporation has been shown to have deleterious effects on neuronal migration during hippocampal development [65]. This suggests that excessive aryl hydrocarbon receptor activation by TCDD may similarly disrupt neurodevelopment, contributing to the pathophysiology of ASD observed in Vietnamese children. In addition to the ability to act on the aryl hydrocarbon receptor, dioxins are also able to act on the thyroid hormone receptor due to their similar molecular reactivity properties to active thyroid hormones. There is increasing evidence for the existence of mutual crosstalk between the signaling pathway mediated by the aryl hydrocarbon receptor and thyroid hormone receptors [66,67]. More evidence in animal experiment studies are available in the Supplementary Materials: File S1.

In a clinical study of patients with ASD, epigenetic differences compared with healthy controls have been observed, including changes in global DNA methylation and hydroxy methylation [68], alterations in genes involved in epigenetic modifications [69], and altered DNA methyltransferase expression [70]. Furthermore, epidemiological studies have suggested that dioxin exposure can alter the methylome [71]. For instance, in a study of Swedish elderly adults, high serum PCB126 (a dioxin-like polychlorinated biphenyl) and octachlorodibenzo-p-dioxin were associated with hypermethylation [72]. This raises the possibility that DNA methylation might mediate the effects of dioxin exposure on the developing brain, resulting in the increased risk of ASD.

### 4.4. Limitations

This study had some limitations that should be considered. Most epidemiological studies involving Vietnam War veterans have been designed to compare exposed and unexposed groups to Agent Orange, and none have analyzed the effects of different levels of Agent Orange or dioxin exposure. In addition, veterans are not the only military personnel who served in Operation Ranch Hand and as Army Chemical Corps-trained soldiers; many Vietnam War veterans without assignment to special missions related to herbicide spraying were speculated to be exposed to Agent Orange during the war. These facts made it difficult to quantify the levels of exposure among these veterans.

Another important limitation of the current review is that almost all studies for the Vietnam veterans are comparisons of prevalence of the diseases or neuronal function test results between the veterans with and without Agent Orange exposure based on the history of military service in Vietnam. Only in the study by Barrett et al. (2001) [44] did exposure categories divide Ranch Hand veterans into three categories, the background, low, and high groups, based on quantified serum dioxin levels, although total dioxin concentrations (ppt), and not toxic equivalent per gram fat, was indicated.

In addition, only few studies considered potential confounding factors for neurological diseases in adults, such as having PTSD due to mental stress associated with and without military service during the war, diabetes and hypertension, medication use, and lifestyle (e.g., smoking and drinking habits). If the sample size of the future study is small, matched pair data analysis is the best way, with enough statistical power to avoid confounding factors.

Furthermore, neurological studies using MRIs of adult populations exposed to dioxins, indicating significant alterations in brain regional volumes, have been conducted only in men, for which the number was limited [18,19,20]. The Seveso Second Generation Health Study involved women, but no effects of TCDD on their neurocognitive functions were observed [73]. However, Eskenazi et al. (2005) [74] investigated these women and reported an increased risk of earlier menopause associated with increased serum TCDD, possibly leading to earlier memory decline, because estrogen deficiency is a recognized risk factor for AD in the general population, and the prevalence of AD is high among postmenopausal women [75]. Future research focusing on exposure of older women to dioxin originating from Agent Orange, to investigate cognitive functions and/or brain regional volumes, is needed, particularly in Vietnam.

## 5. Conclusions and Future Directions

Epidemiological studies evaluating the neurotoxic effects of dioxin exposure in adulthood suggest that dioxin exposure originating from Agent Orange may influence the morphological and functional changes associated with brain aging, although its effects are not conclusive. Studies targeting Vietnam War veterans have suggested significant associations between Agent Orange exposure and increased serum Aβ oligomer levels, dementia diagnosis, brain atrophy, and dementia and/or cognitive disturbance. These findings suggest that dioxins may promote brain aging and the subsequent development of neurodegenerative diseases, particularly AD. Further epidemiological studies with larger sample sizes using objective assessments of brain function and morphology among residents of both sexes are necessary in the areas around former U.S. airbases. In children, we found that perinatal dioxin exposure influenced their neurodevelopment from infancy to childhood and increased traits of neurodevelopmental disorders in both sexes, although the phenotype of the effect differed between boys and girls. We are following up our cohorts and clarifying the effects of dioxin on neurodevelopment in adolescents in the near future. Furthermore, among their fathers, alteration in brain regional volumes and increased social anxiety symptoms associated with perinatal dioxin exposure originating from former U.S. airbases were observed, suggesting that social–emotional impairment is another important aspect of the neurotoxic effects of Agent Orange exposure in adults.

In Vietnam, a 10-year remediation project from 2009 to 2018 has been completed in the second largest former U.S. airbase in Da Nang, where more than 50,000 m^3^ of contaminated soil in landfills that covered an area of 53 hectares was treated [76]. Contaminated soil for clean up in the Bien Hoa airbase is nearly four times the volume of the Da Nang airbase, representing the largest dioxin remediation project ever conducted. Launched in 2019, the dioxin remediation project at the Bien Hoa airbase was conducted, not only to decrease the risk of dioxin exposure to people in the airbase, but also to give back restored land for full use to residents in the communities around the airbase. From 2019 to 2023, over 71,000 m^3^ of contaminated soil was excavated, including 27,888 m^3^ of highly contaminated soil to be secured in short-term storage until treatment and 43,224 m^3^ of lowly contamination soil to be stored in a long-term storage facility [77]. In September 2025, the construction of the in-pile thermal desorption system using thermal conduction heating technology commenced to start the treatment of highly contaminated soil in 2026, which could speed up their project and finish it by 2030 [78]. These suggest that the residential areas around the Bien Hoa former military airbase remain contaminated, and residents are at high risk of dioxin exposure originating from Agent Orange, despite 50 years having passed since the end of the Vietnam War.

Epidemiological studies in adults are crucial to identify populations at risk of neurological diseases, particularly AD, associated with Agent Orange or TCDD exposure in Vietnam. Recently, we have started Vietnam’s first national dementia plan, which will support such epidemiological studies to guide prevention strategies and ensure that adequate care and services are provided to people at increased risk of dementia and their caregivers [79]. Vo et al. (2025) [80] estimated age-standardized rates of incidence and prevalence and important risk factors for AD in all of Vietnam in 2021, using data from the Global Burden of Disease (GBD) 2021 database. However, they have no regional data for Vietnam to investigate area differences in prevalence and risk factors for AD, suggesting the necessity of epidemiological surveys in local areas using high sensitivity methods for AD screening. Currently, our new projects are underway to develop portable near-infrared spectroscopy-based cortical function assessment methods alongside simple cognitive function assessment methods. These methods will be useful for the objective assessment and screening of people with dementia at local public health centers in developing countries such as Vietnam, and they will also be useful for future epidemiological studies.

## Data Availability

No new data were created or analyzed in this study. Data sharing is not applicable to this article.

## Supplementary Materials

The following supporting information can be downloaded at: https://www.mdpi.com/article/10.3390/toxics14050419/s1, File S1: Animal experiment evidence to support epidemiological evidence. References [62,81,82,83,84,85,86] are cited in the supplementary materials.
