# Supplementary material for: Associations Between Neurological Diseases and Dioxin Exposure Originating from Agent Orange in Vietnam: A Narrative Review
Source: Toxics. 2026 May 11;14(5):419. doi: 10.3390/toxics14050419 (PMC13211350; doi:10.3390/toxics14050419)
Supplement: Supplementary file 1 [file toxics-14-00419-s001.zip › toxics-4207975-supplementary.pdf]

Supplementary Materials (S1): Animal experiment evidence to support epidemiological evidence

Although experimental studies using animal models exposed to Agent Orange are rarely found, some studies investigate the effects of 2,4-dichlorophenoxyacetic acid (2,4-D) exposure during the early postnatal period on rat offspring. Rosso et al. (1997) [81] reported significantly reduced brain weight, protein and DNA levels, as well as body weight on the 25th day in pups with higher dose and longer treatment, suggesting a delay in brain development [81]. Duffard et al. (1996) [82] reported a myelin deficit in the brain of rat pups exposed to 2,4-D through maternal milk during the period of rapid myelination (from the 15th to the 25th postnatal days). Among them, the alterations in myelin lipid composition were associated with white matter fiber loss in some brain regions such as the corpus callosum and entorhinal cortex. Also, in offspring rats exposed to 2,4-D during the perinatal period, abnormal behaviors, including delay of the ontogeny of righting reflex and negative geotaxis accompanied by motor abnormalities, stereotypic behaviors (excessive grooming and vertical head movements), and hyperactivity in the open field, were observed (Bortolozzi et al. 1999) [83]. These results suggest that exposure to Agent Orange, including 2,4-D, during the perinatal period or postnatal period, influences brain development and induces behavioral abnormalities in rat offspring.

In animal studies, 2,3,7,8-tetrachlorodibenzo-p-dioxin (TCDD) exposure is commonly used, because TCDD is the most toxic and abundant dioxin congener of Agent Orange. Particularly, effects of low dose TCDD exposure during the perinatal period on behavior and brain in mice were investigated, and impairments of executive function and abnormality of social behavior were reported among them (Endo et al. 2012) [84]. They also found hypoactivation of the medial prefrontal cortex (mPFC) and hyperactivation of the amygdala which were shown by the immunohistochemistry of Arc, suggesting imbalanced mPFC-amygdala activation accompanied with higher brain functions. These results suggested that TCDD may induce brain regional abnormality, which is often found in children with ASD. Sha et al. (2021) [85] reported increased movement ability, novelty exploration, and certain anxiety-related behaviors, indicating hyperactivity in the female offspring mice perinatally exposed to TCDD on postnatal day 68. These hyperactivity-like behaviors were accompanied by the upregulation of certain genes associated with cholinergic neurotransmission or synaptogenesis in their brain, suggesting that TCDD may enhance cholinergic neurotransmission to induce hyperactivity among them.

Adulthood TCDD exposure also induces behavioral abnormalities; impairment of contextual fear memory was found in TCDD-exposed mice, which showed reduced

neurogenesis in the hippocampal dentate gyrus and function (memory) (Latchney et al. 2013) [86], which implies that the hippocampus is a tissue vulnerable to TCDD. These results suggest hippocampal dysfunction associated with adulthood TCDD exposure, which may lead to the development of dementia reported in the Vietnam War veterans as reported in the epidemiological study. In another mice study, Debler et al. (2024) showed depression-like behavior in mice exposed to TCDD [62].

References:

62. Debler, R.A.; Gallegos, P.L.; Ojeda, A.C.; Perttula, A.M.; Lucio, A.; Chapkin, R.S.; Safe, S.; Eitan, S. TCDD (2,3,7,8-tetrachlorodibenzo-p-dioxin) induces depression-like phenotype, *Neurotoxicology* **2024**, *103*, 71-77.

81. Rosso, S.B.; Di Paolo, O.A.; Evangelista de Duffard, A.M.; Duffard, R. Effects of 2,4-dichlorophenoxyacetic acid on central nervous system of developmental rats. Associated changes in ganglioside pattern. *Brain Res* **1997**, *769(1)*, 163–167.

82. Duffard, R.; Garcia, G.; Rosso, S.; Bortolozzi, A.; Madariaga, M.; di Paolo, O.; de Duffard, A.M.E. Central nervous system myelin deficit in rats exposed to 2,4-dichlorophenoxyacetic acid throughout lactation. *Neurotoxicol Teratol* **1996**, *18(6)*, 691–696.

83. Bortolozzi, A.A.; Duffard, R.O.; Evangelista de, Duffard, A.M. Behavioral alterations induced in rats by a pre- and postnatal exposure to 2,4-dichlorophenoxyacetic acid. *Neurotoxicol Teratol* **1999**, *21(4)*, 451–465.

84. Endo T.; Kakeyama,M.; Uemura, Y.; Haijima, A.; Okuno, H.; Bito, H.; Tohyama, C. Executive function deficits and social-behavioral abnormality in mice exposed to a low dose of dioxin in utero and via lactation. *PLoS One* **2012**, *7,* e50741.

85. Sha, R.; Chen, Y.; Wang, Y.; Luo, Y.; Liu, Y.; Ma, Y.; Li, Y.; Xu, L.; Xie, H.Q.; Zhao, B. Gestationa and lactational exposure to 2,3,7,8-tetrachlorodibenzo-p-dioxin in mice: Neurobehavioral effects on female offspring. *Sci Total Environ* **2021**, *752*, 141784.

86. Latchney, S.E.; Hein, A.M.; O'Banion, M.K.; DiCicco-Bloom, E.; Opanashuk, L.A. Deletion or activation of the aryl hydrocarbon receptor alters adult hippocampal neurogenesis and contextual fear memory. *J Neurochem* **2013**, *125*, 430–445.
